# Editorial: Functions of Nitric Oxide in Photosynthetic Organisms

**DOI:** 10.3389/fpls.2022.877438

**Published:** 2022-03-29

**Authors:** Natalia Correa-Aragunde, Noelia Foresi, Christian Lindermayr, Marek Petřivalský

**Affiliations:** ^1^Instituto de Investigaciones Biológicas-Consejo Nacional de Investigaciones Científicas y Técnicas (CONICET), Universidad Nacional de Mar del Plata, Mar del Plata, Argentina; ^2^Institute of Biochemical Plant Pathology, Helmholtz Zentrum München, München, Germany; ^3^Department of Biochemistry, Faculty of Science, Palacký University in Olomouc, Olomouc, Czechia

**Keywords:** nitric oxide, photosynthetic organisms, nitrosation, nitric oxide synthase, GSNO reductase, alternative oxidase (AOX), nutrient use efficiency (NUE), redox signaling

More than 40 years of research consolidate nitric oxide (NO) as a signal molecule required in a broad range of physiological processes in plants. This redox-active molecule participates in a myriad of processes including plant development, abiotic stresses and plant-microbe interactions including plant immunity recently reviewed in dedicated articles (Kolbert et al., 2019; Gupta et al., 2020; León and Costa-Broseta, 2020). In this collection, we have gathered contributions from scientists working in diverse aspects of NO biology in photosynthetic organisms in an attempt to spur new collaborations and accelerate science in this field. This Research Topic, Functions of NO in Photosynthetic Organisms, compiles 1 hypothesis and perspectives, 1 opinion article and 5 original research articles, from different countries, that span the research field and give insight into ongoing novel topics in NO plant biology.

New studies and innovative ideas highlight the importance of NO homeostasis. Zafari et al. demonstrate that overexpression of alternative oxidase (AOX) gene augments NO release under hypoxia, while knock down of *AOX* diminishes NO. AOX participates in the phytoglobin-NO (Pgb1-NO) cycle which can contribute to ATP generation under hypoxia. The amino acid content increased in plants subjected to hypoxia, being higher in the *AOX* overexpressor and lower in the knock down plants compared to the wt. During hypoxic conditions, the Pgb1-NO cycle is highly active, favoring an accumulation of amino acids, which contribute to maintaining carbon, nitrogen, and energy metabolism. Regarding this, nutrient recycling is essential to maximize its efficient use and to deal with environmental situations that limit its availability or potential shortages. NO influences many processes underlying traits related to the nutrient use efficiency (NUE), mainly P, K and N. Buet et al. present an opinion article that puts on the board the complexity of the evaluation of the NO impact on NUE in plants. The authors concern the occurrence of unwanted collateral effects derived from NO manipulation in plants. This opinion proposes some priorities to take into account in the research agenda of plant nutrition leading to an increase of NUE.

NO-dependent post-transcriptional modifications (PTMs) remain a hot topic in plant NO biology and are still of great importance for characterization of NO signaling function. Protein S-nitrosation, the covalent binding of NO to the reactive thiol group of protein cysteine residues, is a post-translational signaling mechanism of NO, which affects the activity, binding properties, stability or subcellular localization of proteins. Treffon et al. performed a quantitative proteomic analysis with S-nitrosoglutathione reductase (GSNOR) null mutant to identify novel components in NO homeostasis in plants. Interestingly, a novel group of proteins belonging to the aldo-keto reductase (AKR) protein superfamily was found to be upregulated in *gsnor* mutants. Results show that similarly to animal cells, AKRs catalyze an NADPH-dependent GSNO and SNO-CoA degradation that contributes to the control of S-nitrosothiol levels in plant cells. This paper thus reveals specific members of the plant AKR family might play an important complementary role to GSNOR in the regulation of NO homeostasis in plants. Moreover, two full papers in this research collection focus on the action of NO through S-nitrosation of target proteins. The work presented by Terrile et al. provides consistent evidence of NO-dependent regulation of SCF^TIR1^ and SCF^COI1^ E3 ligase complexes impacting signaling of auxin and jasmonic acid (JA), respectively, in Arabidopsis. S-nitrosation of Arabidopsis SKP1 protein (ASK1) at Cys118 enhances ASK1-COI1 protein-protein interaction. In addition, overexpression of non-nitrosable *ask1* mutant protein impaired the activation of JA-responsive genes illustrating the functional relevance of this redox-mediated regulation in planta. In another work of this collection, Nicolas-Frances et al. analyzed S-nitrosation of *Arabidopsis thaliana* Protein Tyrosine Phosphatase 1 (AtPTP1), a tyrosine-specific PTPase, which acts as repressor of H_2_O_2_ production and regulates the activity of MPK3/MPK6 MAPKs by direct dephosphorylation. They demonstrated that S-nitrosation of AtPTP1 at Cys265 inhibits its activity and may protect AtPTP1 from irreversible oxidation exerted by H_2_O_2_ (Nicolas-Frances et al.). These results provide evidence that S-nitrosation is a widely distributed PTM that affects not only the activity of the modified enzyme but also modulates complex hormonal and redox signaling.

An intriguing aspect that still needs to be clarified in higher plants are the enzyme sources of NO production. NOS-like activities have been reported, although there are no mammalian NOS homologs in terrestrial plant genomes published to date. However, several NOS homologs have been identified in algal genomes and transcriptomes. These results raise the question of the importance of the presence of NOS and its molecular diversity in algae. Chatelain et al. hypothesize that structural analysis of the of two NOS proteins from *Klebsormidium nitens*, a model alga in the study of plant adaptation to terrestrial life, as well as the identification of interacting partner proteins might allow a better understanding of the functioning of NOS and a deeper insight into NO signaling. The KnNOS1 sequence contains canonical NOS signatures, while the KnNOS2 sequence includes a globin domain in the C-terminus. An *in-silico* approach performed in this work highlights putative NOS interacting partners in the genome of *K. nitens*, which will be further analyzed *via* pull-down assays. Finally, all these results will allow a better understanding of the molecular diversification of NOS in different physiological contexts (Chatelain et al.). Likewise, *Chlamydomonas reinhardtii* is an excellent alga model species for studying NO functions in biological processes in this phylogenetic clade since the genome sequence and molecular tools are available. The metabolic shift and the following acclimation processes of *C. reinhardtii* to a NO burst were examined by transcriptomic analyses (Kuo and Lee). The authors show that the main processes activated by a NO burst are the modulation of ROS signaling pathways together with the induction of the antioxidant defense genes, the reduction of NO levels and the modulation of nitrogen and sulfur uptake. The study of NO sources and the biological function of NO in algal systems may allow us to transfer and/or open new lines of research in higher plants.

Taken together, this collection of articles presents an overview of new interesting lines of research and provide insight into the different exciting topics that increases our understanding of NO functions in photosynthetic organisms. Deepening the efforts that allow to investigate how NO production is controlled and perceived by plants will fill the gaps in our understanding of plant NO homeostasis. In this sense, detection of NO by the new generation of biosensors in plants would be pleasantly welcomed by the plant biology community. Moreover, it would be also desirable to apply our knowledge of NO functions in crops other than model plants in the way to improve crop yield and stress resistance, especially under stressful and climate-changing conditions in the near future. This might include research on application of NO-releasing nanomaterials as well as on NO-dependent genetic and epigenetic mechanisms.

## Author Contributions

All authors listed have made a substantial, direct, and intellectual contribution to the work and approved it for publication.

## Conflict of Interest

The authors declare that the research was conducted in the absence of any commercial or financial relationships that could be construed as a potential conflict of interest.

## Publisher's Note

All claims expressed in this article are solely those of the authors and do not necessarily represent those of their affiliated organizations, or those of the publisher, the editors and the reviewers. Any product that may be evaluated in this article, or claim that may be made by its manufacturer, is not guaranteed or endorsed by the publisher.
